# BIOPHYSICAL PROPERTIES OF SUBTHRESHOLD RESONANCE OSCILLATIONS AND SUBTHRESHOLD MEMBRANE OSCILLATIONS IN NEURONS

**DOI:** 10.1142/S0218339016500285

**Published:** 2016-12-07

**Authors:** BABAK V-GHAFFARI, M. KOUHNAVARD, T. KITAJIMA

**Affiliations:** * Departments of Neuroscience, Cell Biology and Physiology, Wright State University, Dayton, OH, USA; † Malaysia-Japan International Institute of Technology, UTM, Kuala Lumpur, Malaysia

**Keywords:** Subthreshold Membrane Oscillation, Subthreshold Resonance Oscillation, Stellate Cells, Equivalent RLC Circuit, Biophysical Model, Conductance-Based Model

## Abstract

Subthreshold-level activities in neurons play a crucial role in neuronal oscillations. These small-amplitude oscillations have been suggested to be involved in synaptic plasticity and in determining the frequency of network oscillations. Subthreshold membrane oscillations (STOs) and subthreshold resonance oscillations (SROs) are the main constituents of subthreshold-level activities in neurons. In this study, a general theoretical framework for analyzing the mechanisms underlying STOs and SROs in neurons is presented. Results showed that the resting membrane potential and the hyperpolarization-activated potassium channel (*h*-channel) affect the subthreshold-level activities in stellate cells. The contribution of *h*-channel on resonance is attributed to its large time constant, which produces the time lag between *I_h_* and the membrane potential. Conversely, the persistent sodium channels (Nap-channels) only play an amplifying role in these neurons.

## 1. Introduction

The parahippocampal region is extensively known as the central organization of neural processes for memory and learning. In this region, the entorhinal cortex (EC) is the interface between the hippocampus and the neocortex, which generally transfers information from spiny stellate cells (SCs). The electrophysiological properties of SCs are identified by 4 Hz to 18 Hz subthreshold membrane oscillations (STOs) following subthreshold stimuli.^1,2^ These STOs are believed to be important contributors to the hippocampal theta rhythm.^3^ STOs are small-amplitude oscillations (maximum amplitude of approximately 10mV) of membrane potential that are closely related to the synchronous rhythmic activities in many neurons, which are associated with crucial functions of the nervous system, such as perception, ^4^ learning,^5^ and awareness.^6^ In the subthreshold-level activities in neurons, there is another concept that is intimately related to STOs. This concept is resonance, which indicates that such neurons demonstrate frequency selectivity. The property of frequency selectivity of neurons is defined as a tendency of a neuron to generate subthreshold resonance oscillations (SROs), which are peaks in the impedance amplitude, in response to an oscillatory input current with changing frequency at a nonzero (resonant) frequency.^7^ The resonance phenomenon was first reported in myelinated nerves by Hermann.^8^ Since then, this phenomenon has been observed in many excitatory and inhibitory neurons, such as inferior olive,^9^ neocortex, ^10^ thalamus,^11^ hippocampal CA1 area,^8,9^ and EC.^12,13^

The previous work of the authors showed that the equivalent RLC circuit models of neurons are powerful tools to determine the contributions of ion channels to the resonant properties of neurons.^14^ According to electrical circuit theory, resonance is a combination of components with low-pass (RC) and high-pass (RL) filter properties. By using fluctuating sinusoidal (chirp or ZAP stimulus) current stimuli,^10^ it was shown that the passive membrane properties of neurons, which do not sensitively depend on the activity of the neuron, is responsible to low-pass filtering in neurons.^14–16^ On the other hand, some ion channels with large time constants, which are called resonators, were considered to play a significant role in high-pass filtering and oppose the changes in membrane potential. Therefore, the combination of these types of ion channels and passive membrane properties may underlie the resonant properties of neurons.^17,18^

Many previous experimental and theoretical studies have investigated different types of oscillatory behavior in SCs, such as STO,^1,18,19^ spiking,^2,20,21^ and mixed-mode oscillations.^22–24^ However, the mechanisms underlying these oscillatory activities in SCs are still not well-understood. The subthreshold-level activities in SCs emerge from the interactions between the slow hyperpolarization-activated potassium current (*I_h_*) and the persistent noninactivating sodium current (*I*_NaP_).^25,26^ The linear and nonlinear mechanisms responsible for the nature of the oscillatory behavior of SCs may likely transform the information that SCs pass to the hippocampus. The subthreshold-level oscillatory behavior of SCs was shown to be closely related to the linear dynamic properties of the membrane that emerged during the injection of a small magnitude of stimuli.^27^ Therefore, the linearized model (equivalent RLC circuit model of the biophysical model) can be a powerful tool to analyze the subthreshold-level activities in neurons.

In this study, the mechanisms underlying the resonant properties (SROs) and STOs in SCs are investigated. In particular, this study is focused on the contributions of membrane potential, *I*_NaP_, and *I_h_* to the SROs using the equivalent RLC circuit model, along with the examination of the relationship between SROs and STOs using the biophysical modeling simulations. The methods used in this study can also be applied to the biophysical model of other neurons to analyze the mechanisms underlying their subthreshold-level activities.

## 2. Methods

In this research, we use the single compartment biophysical model (conductance-based model) of SCs in layer II EC that was introduced by Acker *et al.*^2^ This model was obtained by the measurement performed by previous studies.^28^ It includes input current (*I*_inp_), passive leak current (*I*_leak_), persistent sodium current (*I*_NaP_) and slow hyperpolarization-activated potassium current (*I_h_*). The time evolution of the membrane potential (*V* ) is given by

(2.1) $$C\frac{dV}{dt}=I_{\text{inp}}-I_{h}-I_{\text{NaP}}-I_{\text{leak}},$$

where *C* is the membrane capacitance (*μ*F/cm^2^). *V* and *t* are the membrane potential (mV) and time (ms), respectively. *I*_inp_ is a time-dependent input current. *I*_leak_ = *g*_leak_(*V* − *E_L_*), and *I_j_* (*j* = NaP and *h*) are the transmembrane ionic currents of the form

(2.2) $$I_{j}=g_{j}(V-E_{j}),$$

where *E_j_* are reversal potential, and *g_j_* are the voltage-dependent conductance variable. *g_j_* is proportionate to the product of maximum conductance density (*ḡ_j_* in (mS/cm^2^)) and probabilities of channels activation (*m*) and inactivation (*h*). For *h* and NaP channels, the voltage-dependent conductance variables are *g_h_* = *ḡ_h_*(0.65*m_hf_* + 0.35*m_hs_*) and *g*_NaP_ = *ḡ*_NaP_*m*_NaP_, respectively. The gating variables obey a first-order differential equation as

(2.3) $$\frac{{dm}_{i}}{dt}=\frac{m_{i\infty }(V)-m_{i}}{\tau_{i}(V)}\;\;\;(i=hf,hs,\text{Nap}).$$

The definitions of *m_i∞_*(*V* ) and *τ_i_*(*V* ) (*i* = *hf*, *hs*, Nap) are given in Appendix A. The values of parameters are: *ḡ*_NaP_ = 0.5, *ḡ_h_* = 1.5, *g*_leak_ = 0.5, *E_L_* = −65, *E*_Na_ = 55, *E_h_* = −20, *C* = 1. We call the situation as control condition when these values are used in simulations. To analyze the STO phenomena, we input a simple sinusoidal current, which describes as

(2.4) $$I_{\text{inp}}=I_{\text{app}}\sin(2\pi ft),$$

where *I*_app_ and *f* are the amplitude of current (*μ*A/cm^2^) and frequency (Hz), respectively. On the other hand, to analyze the SRO phenomena, we input the ZAP current into both biophysical model and its equivalent RLC circuit to examine the voltage response and impedance profile. The ZAP input current is given by

(2.5) $$I_{\text{inp}}=I_{\text{app}}\sin(2\pi f(t)t),$$

where the time-dependent frequency, *f*(*t*), increases from *f*_0_ to *f*_max_ for a total duration *T. f*(*t*) is expressed by

(2.6) $$f(t)=f_{0}+(f_{\max}-f_{0})\;\left(\frac{t}{2T}\right).$$

The impedance profile is obtained as a ratio of the Fast Fourier Transforms (FFTs) of voltage response and input ZAP current given by

(2.7) $$Z(f)=\frac{\mathrm{FFT}(V)}{\mathrm{FFT}(I)},$$

where FFT(*V* ) and FFT(*I*) are the fast Fourier transforms of the voltage response and the input current, respectively. Impedance is a complex quantity with a real part (*Z*_Re_) as resistance and an imaginary part (*Z*_Im_) as reactance. Impedance can also be shown as a vector that comprises magnitude (*|Z*(*f*)*|*) and phase (*φ*(*f*)) as a function of frequency,^14^ which is described as

(2.8) ∣Z(f)∣=(ZRe)2+(ZIm)2,

(2.9) φ(f)=tan-1(ZImZRe).

We also consider the total inductive phase (*φ_L_*). It is the positive area under the inductive region of impedance phase profile, which is described as

(2.10) $$\varphi_{L}=\int_{\varphi (f)>0}\varphi (f)df,$$

where integration is done while the phase *φ_L_ >* 0. Following our previous study, the frequencies below 0.1Hz are removed in both magnitude and phase profiles to avoid irregular distortions of results at low frequencies.^14^ Simulations are performed in MATLAB implementation of the numerical solution method based on the Runge–Kutta fourth-order method. Our results showed that the impedance magnitude (IM) of the biophysical model is associated with noise caused by software restrictions and errors. These errors are removed by using the local regression method.^14^

**Fig. 1:**
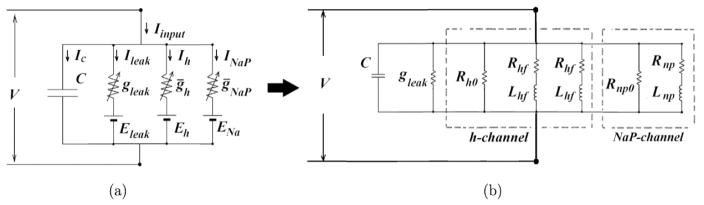
Biophysical model and its equivalent RLC circuit model for SCs. (a) The biophysical model of SCs is a single compartmental neuronal model with *h* and NaP channels. *V* and *C* are the membrane potential and capacitance, respectively. *g*_leak_, *g_h_* and *g*_NaP_ are the maximal conductances for leak, *h* and NaP channels, respectively. *E_L_* is the reversal potential. *E_h_* and *E*_Na_ are equilibrium potentials for *h* and NaP channels, respectively. *I*_inp_ is the synaptic input current. (b) The equivalent RLC electrical circuit model of SCs. The values of resistance and inductance elements for control condition are given in our previous work.^31^

Figure 1 illustrates both biophysical model and its equivalent RLC circuit model for SCs. The detailed explanation of obtaining the equivalent RLC circuits from the linearization of biophysical model are presented in author’s previous studies.^14,29^

**Fig. 2:**
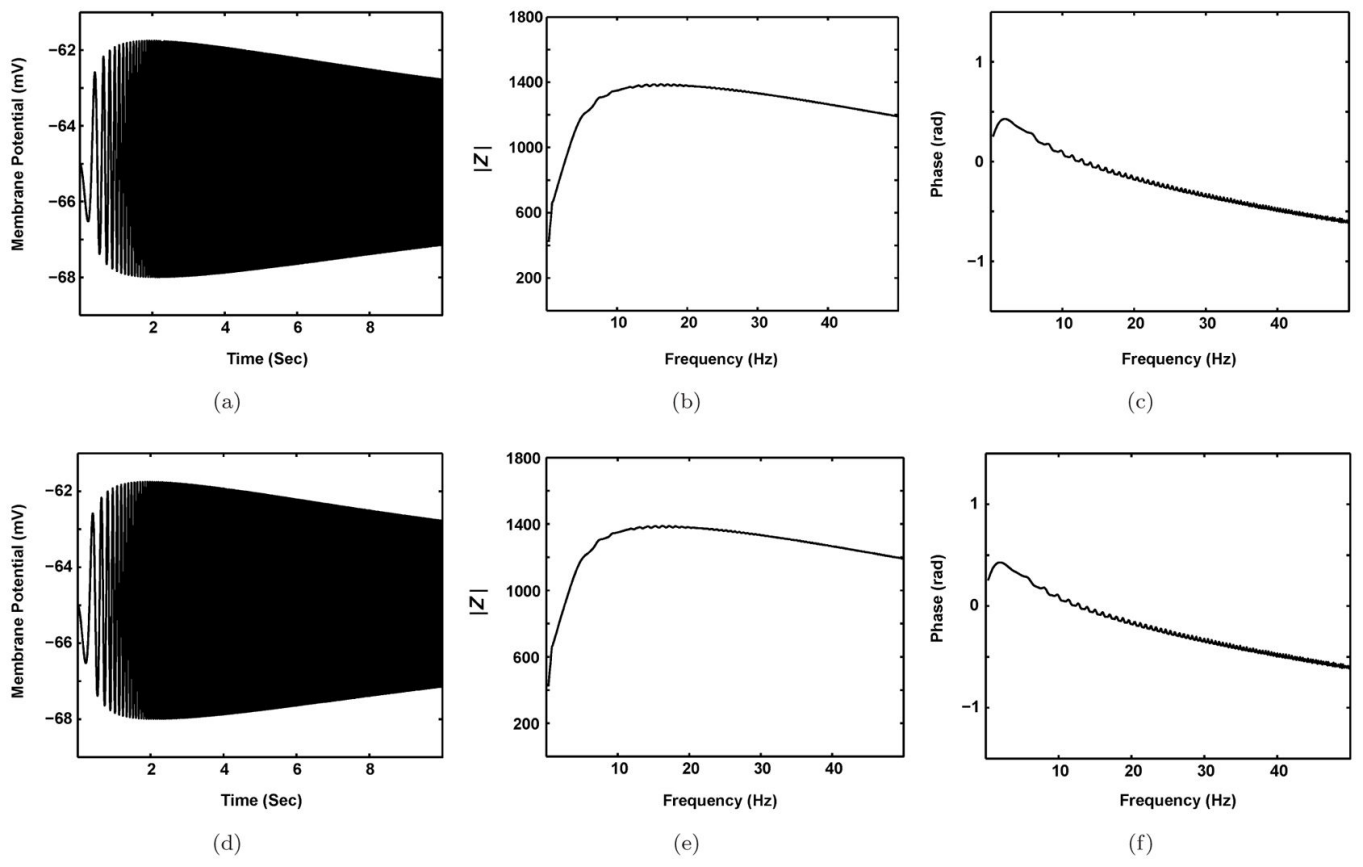
Comparison between the biophysical model and its equivalent RLC circuit model for SCs. The voltage response (a) and impedance profile (amplitude (b) and phase profile (c)) of biophysical model are similar to the voltage response (d) and impedance profile (amplitude (e) and phase profile (f)) of equivalent RLC circuit model. The results obtained under the control condition.

It was shown that if the behavior of the equivalent RLC circuit model in the subthreshold region closely resemble that of the biophysical model, then this circuit can be used to fully analyze the mechanisms underlying subthreshold-level activities in neurons.^14,29^ Also, it should be examined that whether the equivalent RLC circuit model is a good approximation of the biophysical model. To this end, the voltage response and impedance profile of both models in response to the ZAP input are investigated. As shown in Fig. 2, the equivalent RLC circuit model accurately describes the resonant properties of biophysical model of SCs.

## 3. Results and Discussion

The main scope of this study is to present a comprehensive theoretical framework to understand the mechanisms underlying the SRO and STO phenomena in SCs. Therefore, this section is divided into two parts. In the first part, the resonant properties (SROs) of neurons and the effects of ion channels on these properties are investigated through the equivalent RLC circuit model. The main features of resonance, such as resonance frequency, phase profile, and impedance profile, are also explored. In the second part, the effects of ion channels on STO are investigated through the biophysical model. This investigation helps us to determine the contribution of various ion channels to the specific value of membrane potential in STOs.

### 3.1. Resonance

**Fig. 3:**
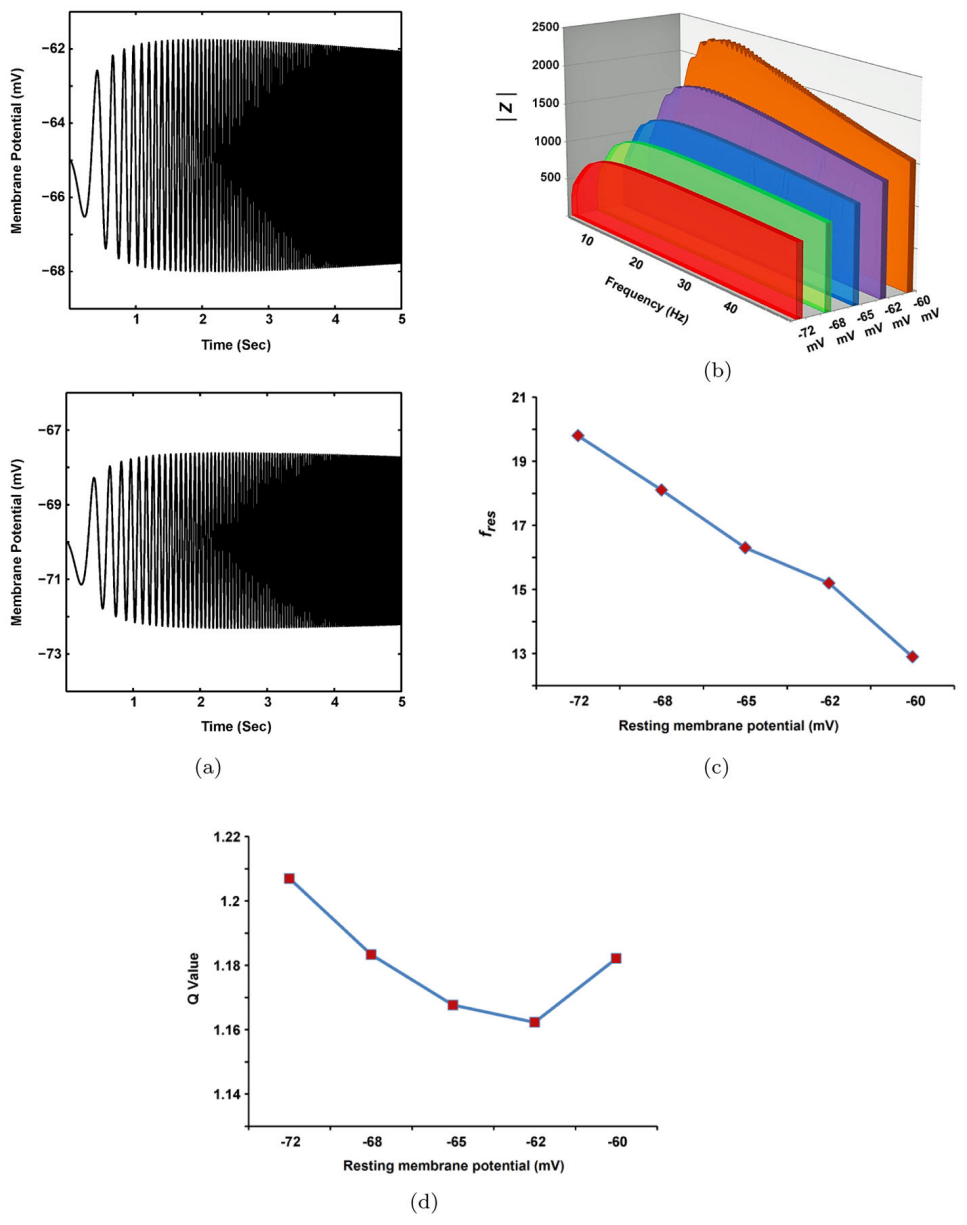
The effect of changing resting membrane potential in the resonant properties of biophysical model of SCs. (a) The voltage response for resting membrane potential at the control condition (upper trace), *V* = −65mV, and hyperpolarized value (lower trace), *V* = −70mV. (b) Three-dimensional plot of impedance amplitude profile (*|Z|*) at different resting membrane potentials. (c) Resonance frequency plotted as a function of membrane potential. (d) *Q*-values (strength of resonance) plotted as a function of membrane potential. For *Q* = 1, there is no resonance in model.

In the first step of analyzing the resonant properties (SRO), the biophysical model of SCs is used to evaluate the roles of resting membrane potential in resonance. The selection of the biophysical model facilitates the comparison between the current results and the results obtained from previous experimental studies. In the second step, the equivalent RLC circuit model is used to investigate in depth the contributions of ion channels to the resonance phenomena in SCs. Figure 3 illustrates the roles of resting membrane potential in the resonant properties of SCs. As shown in Fig. 3(a, lower trace), the maximum magnitude of voltage response decreased and transferred to the higher frequency value in response to the hyperpolarization of resting membrane potential. This decrease is related to the half-activation voltage, the time constant of *I_h_*, and the membrane time constant, which is also shown by the 3D plot of the impedance amplitude (*|Z|*) curves for different values of the resting membrane potential (between −72mV and −60mV), as shown in Fig. 3(b). A comparison of *|Z|* for different values of the resting membrane potential shows that the depolarization of the resting membrane potential increases the maximum amplitude of *|Z|* and transfers the resonance frequency (the frequency in which the maximum amplitude of *|Z|* occurs) to the smaller value ranges (Fig. 3(c)). All of the preceding results are consistent with previous experimental results.^13,26^ Figure 3(d) shows the effect of changing the resting membrane potential on the *Q* value, which is a valuable indicator used to quantify the strength of resonance. The *Q* value implies the sharpness of the impedance curve (*|Z|*) around the resonance frequency, which is obtained by the ratio between maximum impedance and impedance at *f* = 0.5Hz.^14^ The results showed that the suitable quantitative criterion of the *Q* value is *Q ≥* 1.01, which indicates that the maximum impedance should be at least 1% higher than the minimum impedance (*|*(0.5)*|*) for the model to demonstrate resonant properties.

As shown in Fig. 3(d), the resonant properties of SCs are closely related to two membrane potential ranges, such that resonance is prominent during the depolarization of membrane potential (e.g., the *Q* value is 1.18 near −60mV) and reduces to the minimum value by approaching the resting membrane potential in the control condition (−65 mV). The resonant properties of SCs also increased from the *Q* value of 1.16 to the *Q* value of 1.21 during the hyperpolarization of the membrane potential (−72mV). This behavior is almost similar to what was observed in CA1 neurons,^19^ which indicates that the *Q* value exhibits the opposite voltage dependence.

Two types of ion channels that contribute to the resonant properties of SCs are the *h*- and NaP-channels.^2^ The contributions of these ion channels to the resonance are investigated by considering the impedance profile of the equivalent RLC circuit model of SCs.

**Fig. 4:**
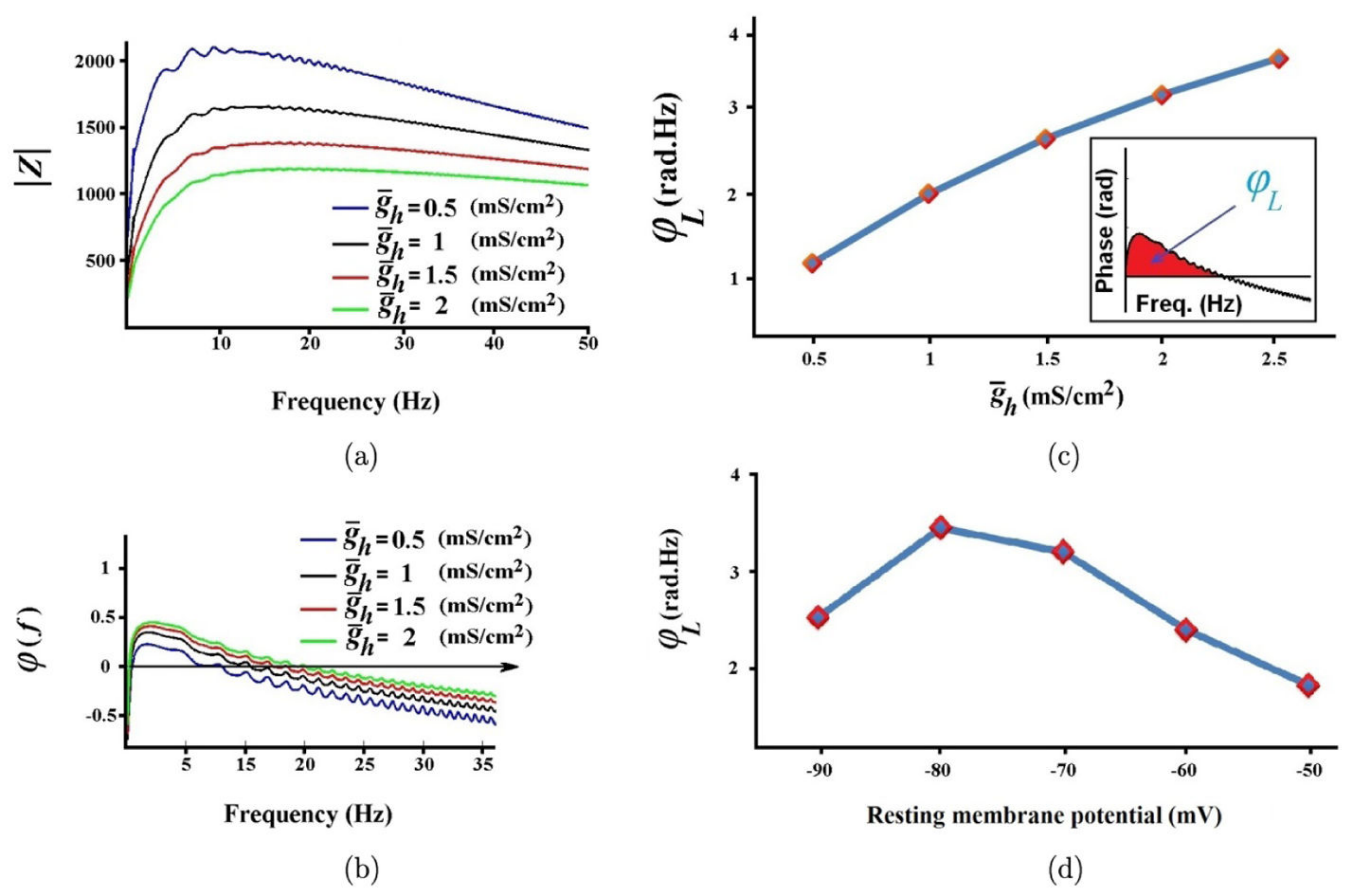
The effect of *h*-channel on impedance profile of the equivalent RLC circuit model of SCs. (a) The impedance amplitude profile shows that increasing of *g_h_* reduces the amplitude of *f*_res_. (b) The impedance phase profiles show that decreasing of *g_h_* reduces the zero-cross frequency (the frequency in which the *φ*(*f*) crosses over zero). (c) The total inductive phase (*φ_L_*) in response to changing of *g_h_*. (d) The total inductive phase (*φ_L_*) in response to the changing of resting membrane potential.

In Fig. 4, the impedance profile diagrams show the effects of changing the maximum conductance of the *h*-channel on the resonant properties of SCs. The results indicate that the increase in *g_h_* shifts the frequency of resonance to the higher frequency ranges (Fig. 4(a)). By contrast, the decrease in *g_h_* changes *|Z|* into the unimodal curve and decreases the frequency of resonance. This behavior is similar to what was reported in PD neurons.^14^ Figure 4(b) illustrates the impedance phase profile (*φ*(*f*)) for different values of *g_h_*. The results show that the voltage is ahead of the input current (*I*_inp_) for low-frequency ranges because of the resonating property of *I_h_*. By contrast, the voltage lags *I*_inp_ for higher frequency ranges attributed to the passive membrane properties.^1^

Figures 4(c) and 4(d) illustrate the alternative definition for the phase–frequency plot. This definition is called the total inductive phase (*φ_L_*), which reliably determines not only the existence of the inductance element in the neuronal model but also qualitatively expresses the size of the inductance element (for more details, see Ref. 1).

As shown in Fig. 4(c), the increase in *g_h_* monotonically increases *φ_L,_* indicating that the inductance element of the model increased. Therefore, the *h*-channel plays the inductive role in subthreshold-level activities in SCs. Moreover, the effect of changing the resting membrane potential on *φ_L_* was investigated. As shown in Fig. 4(d), *φ_L_* has a bell-shaped dependence on membrane potential because of the half-maximum activation of the slow variable of the *h*-channel (approximately −80 mV). The role of the NaP-channel in the resonant properties of SCs was also examined (not shown). The results showed that the NaP-channel only plays the amplifying role and does not affect resonance. This behavior was examined in depth in the previous work of the authors.

### 3.2. Subthreshold oscillation

**Fig. 5:**
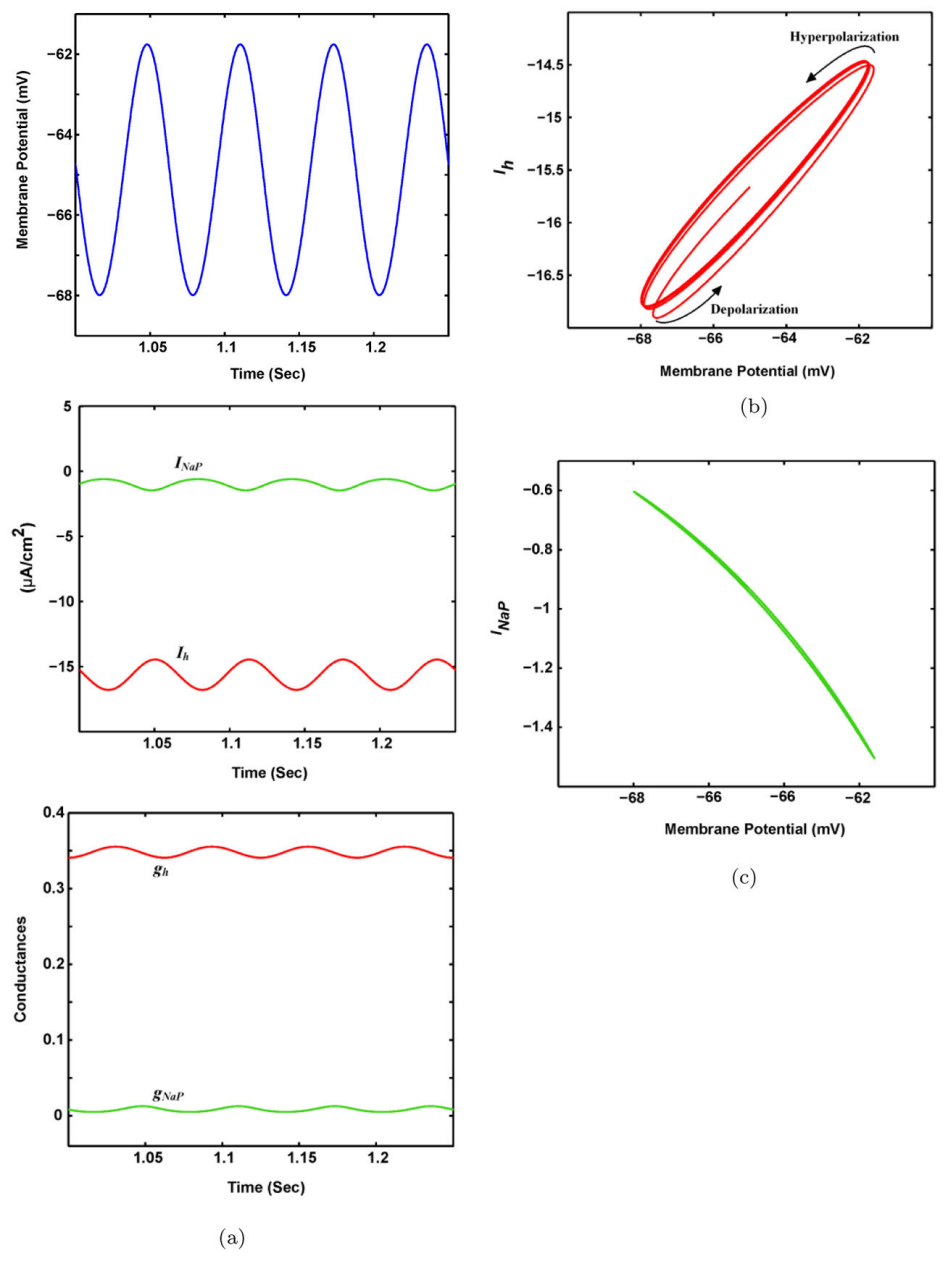
The evolution of SCs dynamics in response to the sinusoidal current. (a) The evolutions of *I*_NaP_ and *I_h_* during STO. Noted that *I*_NaP_ closely follow the evolution of membrane potential. In contrast, *I_h_* lags the membrane potential. The Increase and decrease in *I_h_* correspond to minimum and maximum peaks of the membrane potential, respectively. The phase-plane of the amplitude of *I_h_* (b) and *I*_NaP_ (c) versus the membrane potential. the plot corresponding to *I*_NaP_, demonstrates a straight line. Therefore, the amplitude of *I*_NaP_ changes spontaneously. Plot corresponding to *I_h_* shows the hysteresis. It means, during the downswing and upswing of STO, the amplitude of *I_h_* changes with a significant delay.

In the previous section, the SRO phenomena in SCs are analyzed. However, the STO phenomena should also be analyzed to obtain an in-depth understanding of the resonance behavior in neurons. Therefore, the roles of ion channels in STOs are investigated in this section by injecting the sinusoidal current (Eq. (3.5)) into the biophysical model of SCs. In particular, the investigations of the mechanisms underlying the STO phenomena assist in defining the state of each channel in a specific time. Figure 5(a) illustrates the evolution of membrane potential, conductances, and ionic currents in response to the sinusoidal current in the control condition. For the value of resonance frequency in the control condition, the voltage leads the input current (*I*_inp_) because of the interaction of the slow kinetics of the *h*-channel and the passive membrane properties. Narayanan and Johnson showed that voltage lags input current for resonance frequency in higher ranges (e.g., 60 Hz) because there is no time for the activation or inactivation of the *h*-channel.^1^ In this case, the capacitive component dominates in the generation of STOs in SCs.

The results showed that the hyperpolarization of membrane potential inactivates the NaP-channel, *g*_Nap_ ≈ 0, (Fig. 5(a), lower trace), which in turn decreases the rate of increase of *I*_NaP_ (Fig. 5(a), middle trace).

By contrast, the *h*-channel is slowly activated (*g_h_* increases) during the hyperpolarization of membrane potential (Fig. 5(a), lower trace), which in turn increases *I_h_* (Fig. 5(a), middle trace). *I_h_* notably lags the membrane potential for almost one cycle because of the slow kinetics of the *h*-channel. The slow activation of the *h*-channel corresponds to the large value of its time constant.

During the depolarization of membrane potential, the Nap-channel is rapidly activated, which in turn raises the slope of the *I*_NaP_ increase curve. By contrast, the *h*-channel is slowly inactivated because of its slow kinetics. Therefore, *I*_NaP_ instantaneously changes with the changes in the membrane potential, whereas *I_h_* cannot follow the evolution of the membrane potential, as shown in Fig. 5(a) (middle trace).

*I*_NaP_ and *I_h_* are plotted as a function of membrane potential to better understand this differential behavior (Figs. 5(b) and 5(c)). Notably, the *I_h_* curve exhibits substantial hysteresis, whereas the *I*_NaP_ curve exhibits no hysteresis.

As shown in Fig. 5(b), two different *I_h_* trajectories exist during the hyperpolarizing and depolarizing phases of membrane potential. *I_h_* slowly decreases during the initial part of the depolarizing phase. When the membrane potential is further depolarized, the increase in *I_h_* is expedited. Similarly, *I*_NaP_ slowly increases during the initial portion of the hyperpolarizing phase. This rate of increase is expedited by further hyperpolarizing the membrane potential. The changes of *I*_NaP_ during the hyperpolarizing and depolarizing phases sustain an almost linear relationship with changes in membrane potential. Essentially, the hysteresis observed in the *I_h_* curve is produced by the slow kinetic properties of *I_h_*. This phenomenon delays the feedback mechanism to the membrane potential changes and leads to the sustained oscillatory activity of the membrane.

## 4. Conclusion

Subthreshold-level activities in neurons emerge when the small-amplitude current is injected into the membrane of the neuron. These types of oscillatory behavior are reported to play crucial roles in synaptic plasticity and frequency of neural network oscillations.^1,12,30^ However, the mechanisms underlying these oscillations have not yet been completely understood. The subthreshold-level activities in neurons can be categorized into two main groups, namely, STOs and SROs. In previous studies, these subthreshold-level activities have been shown to be governed by various voltage-dependent ion channels.^1,7,9^ Therefore, investigating the roles of these ion channels in the generation of subthreshold-level activities is crucial. For this purpose, this study examined the roles of ion channels and membrane potential in the STO and SRO phenomena in SCs. Following the prominent work of Lampl and Yarom,^9^ the STO and SRO phenomena in SCs were shown as two manifestations of the same mechanism. The previous study of the authors of this paper showed that the equivalent RLC circuit model obtained by linearizing the biophysical model is a powerful tool to determine the contribution of ion channels to resonance behavior in neurons. In the current study, the results showed that the *h*- and NaP-channels play resonating and amplifying roles in SCs, respectively. Removing *I_h_* notably eliminates the resonance; however, removing *I*_NaP_ only decreases the amplitude of oscillation. The comparison between the results obtained for the STO and SRO phenomena determined that the property of frequency selectivity of SCs emerged because of the diversity of time constants in the model. The time constant of *I*_NaP_ is small; therefore, it can closely follow the evolution of membrane potential. Moreover, *I*_NaP_ is a positive inward current. Therefore, the depolarization of membrane potential results in the fast activation of the NaP-channel, which in turn accelerates the depolarization of membrane potential. Conversely, the time constant of *I_h_* is relatively large. Therefore, *I_h_* cannot follow the evolution of membrane potential; thus, *I_h_* always lags the membrane potential. The *h*-channel is a positive outward current that slowly activates during the hyperpolarizing phase of membrane potential. This property of the *h*-channel produces the negative feedback between *I_h_* and membrane potential, which indicates that *I_h_* opposes the changes in membrane potential. During the hyperpolarizing phase, the *h*-channel slowly activates, which further hyperpolarizes the membrane potential. The depolarization of membrane potential slowly inactivates the *h*-channel. The inactivation of the *h*-channel further depolarizes the membrane potential. These results show that only the *h-*channel contributes to the resonant properties of SCs and the NaP-channel only affects the strengthening or weakening of the resonance amplitude. The effect of changing the resting membrane potential on resonance is also investigated in this study. The results showed that the increase in resting membrane potential indicates the resonant properties of SCs. This behavior is similar to what was reported in PD neurons.^14^ This study provided a theoretical framework for analyzing the STOs and SROs in neurons. Future studies could focus on the investigation of the STOs and SROs in other neurons, such as pyramidal and thalamic neurons. Future works should also clarify the relationship between resonant phenomena in the biophysical model of a single neuron and neural networks. Finally, although this study focused on SC neurons, the methods and simulations also extend to other regions of the nervous system.

## Appendix A

All the activation and inactivation gating variables obey a first-order differential equation defined in terms of voltage-dependent steady-state function (*x_∞_*) or time constant (*τ_x_*), as described in Eq. (2.3). The definitions of *x_∞_*(*V* ) and *τ_x_*(*V* ) functions for each variable as follows.

(A.1) $$m_{hf\infty }(V)=\frac{1}{\left(1+\exp((V+79.2)/9.78)\right)},$$

(A.2) $$\tau_{\mathrm{mhf}}(V)=1+\frac{0.51}{\left[(\exp((V-1.7)/10))+(\exp(-(V+340)/52))\right]},$$

(A.3) $$m_{hs\infty }(V)=\frac{1}{\left(1+\exp((V+71.3)/7.9)\right)},$$

(A.4) $$\tau_{\mathrm{mhf}}(V)=1+\frac{5.6}{\left[(\exp((V-1.7)/14))+(\exp(-(V+260)/41))\right]},$$

(A.5) $$m_{\text{NaP}\infty }(V)=\frac{1}{\left(1+\exp(-(V+38)/6.5)\right)}.$$

The time constant of NaP-channel is *τ_m_*_NaP_ = 0.15.
